# Supplementary material for: Diversity of Intrinsic Frequency Encoding Patterns in Rat Cortical Neurons—Mechanisms and Possible Functions
Source: PLoS One. 2010 Mar 19;5(3):e9608. doi: 10.1371/journal.pone.0009608 (PMC2841633; doi:10.1371/journal.pone.0009608)
Supplement: Appendix S1 — Supplementary material for the main article. (0.79 MB RTF) [file pone.0009608.s001.rtf]

Appendix S1: Mathematical solutions

Analytical solution of integrate-and-fire model. Suppose that initially t = 0, the neuron has just fired and the membrane potential is reset to v(0) = Vrest. Before a spike has occurred, it is easy to get the theoretical solution for this integrate-and-fire model (Eq. 1) with the sinusoidal synaptic current driving force Iapp(t) = C (1 + cos(2ðFt )) when no noise term is presented , and . The solution is 

This expression (Eq. 2) describes the membrane potential for 0 < t < t* and is valid up to the moment of the next threshold crossing, where v(t*) = Vè. After a spike is generated, the membrane potential is reset to Vrest and the integration restarts. Using integration by parts, Eq. 2 can be solved explicitly as 

If time t is infinitely long and no threshold is applied in the system, the limit membrane potential v(t) would lie in the range between

If the threshold the output firing rate would be zero and the corresponding critical value of the input frequency F* can be calculated explicitly by  Therefore, 

When F < F*, periodic spiking is guaranteed to be generated. 

Equivalent ordinary differential equation system and its limit cycle. To explore the dynamical behavior of the system and to show the properties of the model with sinusoidal input signal, we convert the integrate-and-fire model (Eq. 1) into an autonomous dynamical system, by introducing two more variables x and y from the periodicity of the input frequencies. Let 

Excluding the noise term, the ODE system equivalently becomes: 

Because of the periodicity in x and y, a solution can be regarded as a curve winding on a cylinder: x2 + y2 = C2. The limit cycle (a trajectory in phase space having the property that at least one other trajectory spirals into it as time approaches infinity) of this ODE system is not explicit. However, the maximal and minimal values of the limit cycle can be solved for theoretically by assuming that no threshold is applied to the neuron firing model, and that time tends to infinity, where the solution (Eq. 3) will tend to the limit cycle. In this case, the exponential terms in Eq. 3 tend to zero, and the remaining sinusoidal terms remain oscillating: 

According to the properties of the sinusoidal formula, we can set 

so that 

Since we know that  we can find the two optimal points on the limit cycle as  

From Eq. 6, it is not hard to see that the difference between the maximal and minimal values of limit cycle becomes smaller and smaller if F becomes bigger and bigger. In other words, the degree of tilt of the limit cycle decreases as F increases (Fig. 5). 
