# Supplementary material for: Diversity of Intrinsic Frequency Encoding Patterns in Rat Cortical Neurons—Mechanisms and Possible Functions
Source: PLoS One. 2010 Mar 19;5(3):e9608. doi: 10.1371/journal.pone.0009608 (PMC2841633; doi:10.1371/journal.pone.0009608)
Supplement: Appendix S2 — Comparison with a circle mapping model. (0.11 MB RTF) [file pone.0009608.s002.rtf]

Appendix S2: Circle mapping 

Keener et al (1981) carried out an analysis of the response of the integrate-and-fire neuron to oscillatory input, dividing the parameter region into three parts for different dynamical properties:
(I)	Phase locking for a subset T of parameter values, and ergodic behavior on its complement, where meas meas(·) refers to the measure of a set, and TC denotes the complementary of set T.  (TC) ≠ 0.
(II)	Phase locking for almost all parameter values and aperiodic behavior otherwise (meas(TC) = 0)
(III)	Quenching, where firing eventually stops.

The dimensionless version of integrate-and-fire model proposed in their paper is

and u(ô+) = 0 if u(ô) = 1. The parameters used in their model correspond to our model in this way 

Hence, our suprathreshold case where Cã > Vè which leads to a constant output firing rate corresponds exactly to the parameter region II (S > ó) in their paper, where the circle mapping ôN+1 = f(ôN) is a piecewise monotonic function and phase locked almost everywhere except for parameter values on a set of measure zero. When S < ó and B > (ó/S – 1) · , (which is the same as F < F* in our model), where phase locking occurs for all parameters except on a set of measure zero; when S < ó and B < (ó/S - 1) · (which is F > F*), the firing process terminates. 

Furthermore, it is also noted in their paper that the larger B is, the more possible it is to have phase locking. However, in our model, B is fixed at 1, and in this sense phase locking would definitely occur yet may not be as strong as with larger values of B.
